# E-Textile Embroidered Metamaterial Transmission Line for Signal Propagation Control

**DOI:** 10.3390/ma11060955

**Published:** 2018-06-05

**Authors:** Bahareh Moradi, Raul Fernández-García, Ignacio Gil

**Affiliations:** Department of Electronic Engineering, Universitat Politècnica de Catalunya, Terrassa 08222, Barcelona, Spain; raul.fernandez-garcia@upc.edu

**Keywords:** e-textile, metamaterials, transmission line, wearable, split ring resonator

## Abstract

In this paper, the utilization of common fabrics for the manufacturing of e-textile metamaterial transmission lines is investigated. In order to filter and control the signal propagation in the ultra-high frequency (UHF) range along the e-textile, a conventional metamaterial transmission line was compared with embroidered metamaterial particles. The proposed design was based on a transmission line loaded with one or several split-ring resonators (SRR) on a felt substrate. To explore the relations between physical parameters and filter performance characteristics, theoretical models based on transmission matrices’ description of the filter constituent components were proposed. Excellent agreement between theoretical prediction, electromagnetic simulations, and measurement were found. Experimental results showed stop-band levels higher than −30 dB for compact embroidered metamaterial e-textiles. The validated results confirmed embroidery as a useful technique to obtain customized electromagnetic properties, such as filtering, on wearable applications.

## 1. Introduction

Metamaterials (MTMs) have attracted significant attention from the science community since 2000. These artificial structures are usually designed to obtain controllable and inaccessible electromagnetic (EM) or optical properties not found among natural materials. MTM transmission lines have been a subject of intensive research in the last few years. One suitable host transmission line is the microstrip, a class of electromagnetic waveguides consisting of a strip conductor and a ground backplane, separated by a thin dielectric, due to its high compatibility with active devices and excellent balance between cost, size, and characteristic impedance control. Different kinds of sub-wavelength resonators have been researched to achieve selective frequency responses. From the point of view of the effectiveness of these materials, MTMs have been applied to improving microwave systems such as antennas, sensors, or waveguides [1,2,3]. Among them, a split-ring resonator (SRR) is a widely proposed magnetic resonant structure [4,5]. The SRR structure consists of a ring with a gap, which corresponds to an equivalent inductance and capacitance, thus generating an equivalent LC tank. The transmission coefficient of the SRR is minimum at the magnetic resonance frequency, i.e., at the resonance frequency of the LC tank. It is important to properly design the SRR structures in the substrate of the microstrip components because the interaction with the host line depends on the shape, the orientation, and the arrangement of the SRR. In addition, the dimensions of the SRRs also affect the equivalent inductance and capacitance values [6]. Since we can control and optimize the design of microstrip components by using SRRs, it is theoretically feasible to implement such structures in textile substrates in order to optimize the performance of wearable or electronic textile (e-textile) devices. E-textile technique requirements are flexibility, lightweight, low-profile, and compactness. Therefore, microstrip technology is a preferred solution to implement smart textile applications. Moreover, a high level of attachment must be achieved between the metallic layers and the textile substrates, while remaining comfortable to wear and complying with the health regulations regarding specific absorption rate (SAR). Embroidery is the most advanced integration technique for electronic textile substrates because embroidery machines allow repeatability, mass production of garments, and customized designs in terms of thread distribution with a resolution in the order of <1 mm [7]. Textile MTMs have been directly reported in literature for antenna applications [8]. Textiles have been used in composite polymer fiber fabrics with guided mode photonic crystal resonances in the gigahertz band [9], and embroidered copper thread split-ring resonators have been used as a narrow-band solution to reduce the electromagnetic radiation [10]. 

In this paper, a novel wearable e-textile MTM based on embroidered transmission line loaded with a split-ring resonator is presented and its performance is analyzed in terms of frequency control of the textile MTM transmission line for signal propagation applications at ultra-high frequency (UHF) microwave frequencies. The main novelty of the paper is based on the use of textile materials as substrate and metamaterial structure. The controllable electromagnetic properties of proposed structure and the possibility of device flexibility and miniaturizability allows for design of the prototype with improved performance and novel techniques, in comparison with conventional devices. Crucially, in comparison with standard printed circuit-board (PCB), whose fabrication techniques enable straightforward realization of the small conductive structures required as rigid circuit boards, embroidery textile materials pose limitations to the achievable size, conductivity, substrate permittivity, substrate dielectric losses, and thickness of the prototypes. 

The proposed prototypes have been simulated by means of the commercial full 3D electromagnetic CST Microwave Studio 2018 software (CST Company, Darmstadt, Germany). The e-textiles to be tested have been fabricated and analyzed in bandwidths between 1.2–3 GHz in a free space environment. Finally, the coupled and electrical models have been extracted by using the commercial Keysight Advanced Design System 2018 software (Keysight, Santa Rosa, CA, USA). The results of the proposed designs show that e-textile MTMs can be applied in filtering UHF microwave applications with a good level of accuracy. The paper is organized as follows. In Section 2, the geometrical sketch and the proposed design is presented and compared with a conventional e-textile transmission line. Section 3 presents the theoretical circuit analysis of the proposed designs. In Section 4, the modeling of symmetrical SRRs e-textile transmission line is studied. In Section 5, the effect of bending of the prototype was studied. In the last section of the paper, the conclusion is given.

## 2. Metamaterial E-Textile Design and Electrical Circuit Model

**Figure 1:**
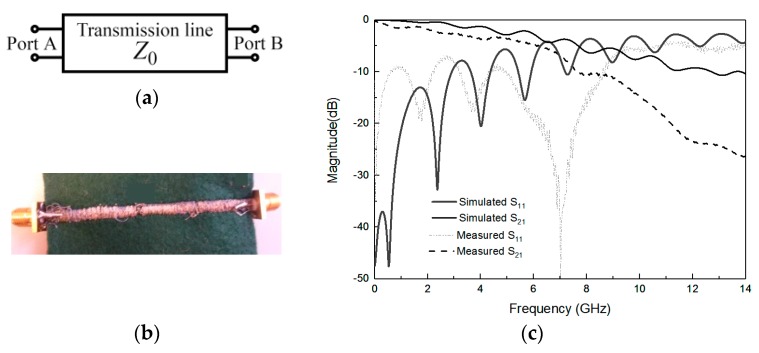
(**a**) The circuit representation of the stitched transmission line. (**b**) Photograph of the embroidered design. (**c**) Simulated and measured S-Parameters of stitched transmission line.

As a first step, a conventional microstrip line has been designed with a 50 Ω characteristic impedance. The substrate was made of felt because of its intrinsic low-loss tangent in comparison with other fabrics [7]. Indeed, in order to determine the substrate dielectric constant and loss tangent of felt substrate, the resonance method based on a split post dielectric resonator (SPDR) measurement has been carried out. The felt fabric was characterized with h = 1 mm thickness, dielectric constant *ɛ*_r_ = 1.2, and loss tangent tan *δ* = 0.0013. The fabric structure was a non-woven structure with a 100% polyester (PES) composition. This fabric had been selected due to its durability and resistance. In fact, these textile substrates are resistant to tearing and humidity, and they offer some key advantages, including durability, chemical moisture resistance, and heat stability. The weight is 211 g/m^2^, and the structure is a double-sided needle punching. The ground plane had been chosen as a homogeneous uniform commercial WE-CF adhesive copper sheet layer (constant thickness t = 35 µm). Figure 1 illustrates the transmission line basic model, the embroidered e-textile (microstrip width W = 5 mm and length L = 77 mm), and the simulated and experimental S-parameters. The insertion losses (S21) and return losses (S11) were tested up to 14 GHz by means of a microwave analyzer, N9916A FieldFox (Keysight, Santa Rosa, CA, USA), operating as a vector network analyzer. At 3 GHz, the maximum frequency limit of UHF, a good matching level for practical applications was achieved, with acceptable losses (lower than 3.5 dB). Losses became higher than 10 dB at frequencies beyond 8 GHz. This effect is explained by the discontinuity of the embroidered stitches, as well as for the thread ohmic losses and the impedance of the transmission line equivalent inductance at higher frequencies. Furthermore, for UHF applications, we determined a maximum attenuation of Att = 0.45 dB/cm, and this microstrip conventional e-textile was considered a test reference. The details of the conductive thread and embroidery process are provided below. 

**Figure 2:**
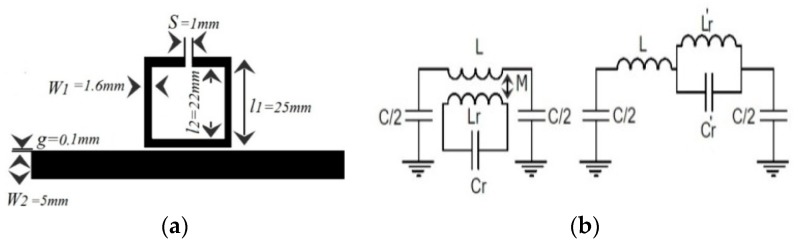
(**a**) Layout of the e-textile transmission line loaded with one SRR. (**b**) Lumped element circuit model that considers magnetic coupling between line and SRR.

In order to control and filter the UHF propagated signal, the conventional e-textile microstrip line can be loaded by using a metamaterial resonator, such as an SRR. The proposed design layout and its lumped element equivalent circuit model are depicted in Figure 2a,b. The dimensions of the proposed design are set to l_1_ = 25 mm, l_2_ = 22 mm, g = 100 µm, s = 1 mm, W_1_ = 1.6 mm (the width of SRR), and W_2_ = 5 mm (the width of the transmission line). Again, the ground plane has been chosen as a homogeneous uniform commercial WE-CF adhesive copper sheet layer (constant thickness t = 35 µm).

With the aim to determine the electrical circuit model, the magnetic wall concept was applied in that model, where the L and C are the line inductance and capacitance, respectively, of the unit cell; Lr and Cr are the inductance and capacitance of the SRR. Finally, M accounts for the magnetic coupling between the line and the ring. The extracted parameters from the method reported in Reference [11] were the following: L = 6.5 nH, C = 3 pF, Cr = 0.3 pF, Lr = 16 nH, M = 0.55 nH.

**Figure 3:**
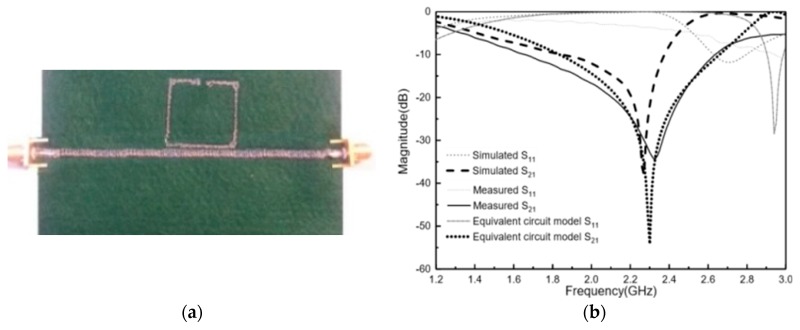
(**a**) Photograph of the embroidered design with satin pattern with 60 % density (top view) and ground implemented with WE-CF adhesive copper. (**b**) S-parameter responses of the electromagnetic (EM) simulation, equivalent circuit model, and measurement of embroidered transmission line loaded with one SRR photograph of the embroidered design.

The proposed e-textile MTM is shown in Figure 3a. The homogeneous layout was converted to a stitch pattern by using the Digitizer Ex software (Version, Elna Company, Genève, Switzerland) for the fabrication process. This software package was used to create the stitch pattern, which was then exported to the embroidery machine (Singer Futura XL550) (SIGNER Company, La Vergne, TN, USA) and stitched. The metamaterial transmission line conductivity was achieved by embroidering high conductive metal threads. The selected conductor yarn was a commercial Shieldex 117/17 dtex two-ply and was composed of 99% pure silver-plated nylon yarn 140/17 dtex with a linear resistance <30 Ω/cm. Due to the mechanical restrictions of the embroidery machine, the pattern was stitched with two types of threads, in which the sulky yarn was used as top thread and the conductive threads were used as bobbin threads. In addition, the default higher thread tension had to be lowered in order to maintain geometrical accuracy. To achieve high geometrical accuracy, we increased the embroidery density to boost surface conductivity and the felt substrate had been chosen to have low embroidery tension and high flexibility.

The proposed design was embroidered with a satin pattern with 60% density. The stitch spacing corresponded to the distance between two needle penetrations on the same side of a column. The density determined the gap between stitches. For narrow columns, stitches were tight, thus requiring fewer stitches to cover the fabric. In areas with very narrow columns, less dense stitches were required because too many needle penetrations can damage the textile sample. The comparison between the theoretical circuit model, full 3D simulation, and the measurement result is shown in Figure 3b. There is good agreement between the simulated, equivalent circuit model, and the measured results. In addition, it was observed that, compared to the conventional SRR, embroidered SRR with the same electrical size provides a wider bandwidth and a stronger resonance. An experimental stop band rejection level of S21 < −30 dB at 2.3 GHz was achieved. This fact demonstrates the effectiveness of the embroidered SRR for frequency propagation filtering in e-textile transmission lines.

## 3. Metamaterial E-Textile Theoretical Circuit Analysis

**Figure 4:**
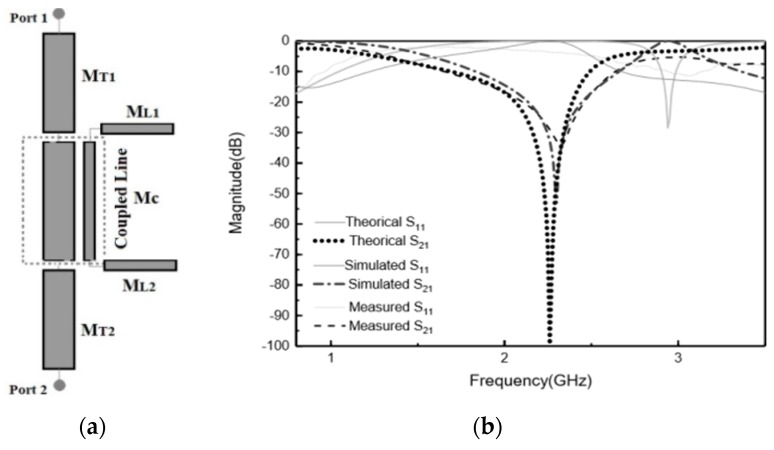
(**a**) Schematic of proposed model of prototype with one embroidered SRR. (**b**) S-parameter responses of the EM simulation, theoretical model, and measurement of embroidered transmission line loaded with one SRR.

We have assessed the theoretical analysis of the circuit describing the proposed MTM e-textile structure, described in Section 2, by means of the technique described in Reference [11] so that this technique could be applied to the embroidered e-textiles that needed testing. In this approach, the inductance and capacitance parameters of asymmetrical coupled lines were determined from the characteristic impedances and effective dielectric constants of the even and odd modes of symmetrical coupled lines. This approach is useful in practice because the even- and odd-mode parameters of symmetrical coupled lines are generally more readily available. As shown in Figure 2a, the MTM e-textile transmission line consists of two coupled lines with widths W_1_ and W_2_, separated by a gap, g, between them. It was assumed that the mutual inductance and capacitance between the lines was the same as that between symmetrical lines of width (W_1_ + W_2_)/2. By using the even- and odd-mode data of coupled symmetrical lines, the mutual inductance and mutual capacitance between the lines was computed, and it was assumed that the self-inductance and capacitance of line one in the presence of line two is the same as if line two had the same width as line one. The coupled transmission line and SRR were characterized through the even and odd characteristic impedance and electric length (*Z_oe_*, *θ_oe_*, and *Z_oo_*, *θ_oo_*, respectively). This implied the coexistence of two different modes with different phase velocities and propagation constants corresponding to the different effective relative permittivities. The even- and odd-mode characteristic impedances, *Z_oe_* and *Z_oo_*, were obtained from the expressions detailed in Reference [12]. The proposed MTM embroidered e-textile was modeled by means of the distributed equivalent circuit model shown in Figure 4a.

This model was described using a single ABCD matrix between the input and output ports. MATLAB software was used for the numerical evaluation of the matrices’ parameters. The internal structure of the overall ABCD matrix, and its component *M_j_* matrices, can be described using the following equations:

(1) $$\left[\begin{array}{cc} A & B\\ C & D \end{array}\right]=M_{T1}.Mc.M_{L1}.M_{L2}.M_{T2}$$

*M_T_*_1,2_ is the ABCD matrix of the line of the ports,

(2) $$M_{T1,2}=\left[\begin{array}{cc} \cos\theta_{T} & jZ_{T}\sin\theta_{T}\\ j\frac{1}{Z_{T}}\sin\theta_{T} & \cos\theta_{T} \end{array}\right]$$

*M_C_* is the ABCD matrix associated to the coupling between the host line and SRR, and

(3) $$M_{C}=\left[\begin{array}{cc} \frac{-Z_{11}}{Z_{12}} & \frac{-Z_{11}^{2}-Z_{12}^{2}}{Z_{12}}\\ \frac{-1}{Z_{12}} & \frac{-Z_{11}}{Z_{12}} \end{array}\right]$$

*M_L_*_1,2_ is the ABCD matrix associated to the SRR line.

(4) $$M_{L1,2}=\left[\begin{array}{cc} \cos\theta_{t} & jZ_{t}\sin\theta_{t}\\ j\frac{1}{Z_{t}}\sin\theta_{t} & \cos\theta_{t} \end{array}\right]$$

The physical dimensions shown in Figure 4a that correspond to the electrical parameters were *Z_T_* = 75.04 Ω, *Z_t_* = 75 Ω, *Z_oe_* = 123.72 Ω, *Z_oo_* = 29.04 Ω, *θ_e_* = 14.17, and *θ_o_* = 13.95, respectively. The result was used to calculate the network parameters of the coupled lines.

(5) $$Z_{12}=Z_{21}=\frac{-j}{2}Z_{oe}.\mathrm{csec}(\theta_{e})+\frac{j}{2}Z_{oo}.\mathrm{csec}(\theta_{o})$$

(6) $$Z_{11}=Z_{22}=\frac{-j}{2}Z_{oe}.\cot(\theta_{e})-\frac{-j}{2}Z_{oo}.\cot(\theta_{o})$$

where $Z_{ij}$ corresponds to the *Z*-matrix coefficient of a two-port network. Therefore, the ABCD matrix of the cascade connection of the two-port network is:

$$\left[\begin{array}{cc} A & B\\ C & D \end{array}\right]=\left[\begin{array}{cc} \cos\theta_{T} & jZ_{T}\sin\theta_{T}\\ j\frac{1}{Z_{T}}\sin\theta_{T} & \cos\theta_{T} \end{array}\right]\left[\begin{array}{cc} \frac{-Z_{11}}{Z_{12}} & \frac{-Z_{11}^{2}-Z_{12}^{2}}{Z_{12}}\\ \frac{-1}{Z_{12}} & \frac{-Z_{11}}{Z_{12}} \end{array}\right]\left[\begin{array}{cc} \cos\theta_{t} & jZ_{t}\sin\theta_{t}\\ j\frac{1}{Z_{t}}\sin\theta_{t} & \cos\theta_{t} \end{array}\right]\;\left[\begin{array}{cc} \cos\theta_{t} & jZ_{t}\sin\theta_{t}\\ j\frac{1}{Z_{t}}\sin\theta_{t} & \cos\theta_{t} \end{array}\right]\left[\begin{array}{cc} \cos\theta_{T} & jZ_{T}\sin\theta_{T}\\ j\frac{1}{Z_{T}}\sin\theta_{T} & \cos\theta_{T} \end{array}\right]$$

The comparison of theoretical, simulation, and measurement results is depicted in Figure 4b. As observed, there was a good agreement between the ABCD-matrix theoretical prediction, the full 3D electromagnetic simulation, and the measured results. Therefore, the coupled presented model describes accurately the frequency behavior of an MTM embroidered e-textile line with one resonator. 

## 4. Metamaterial E-Textile Symmetrical Transmission Line Modelling

**Figure 5:**
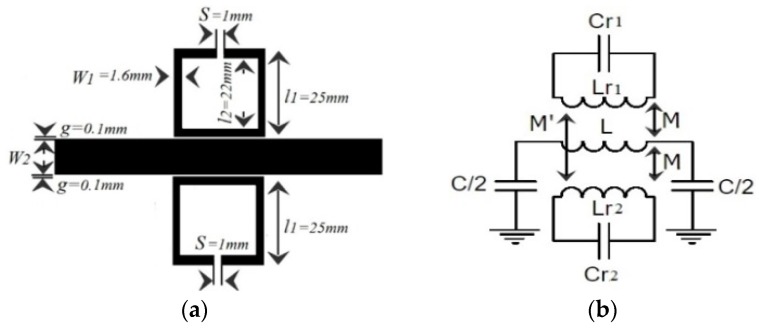

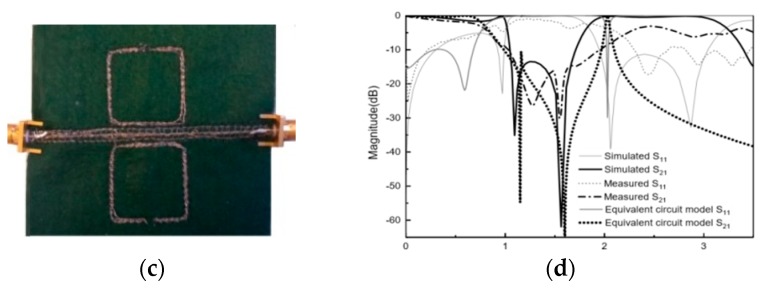
(**a**) Layout of transmission line loaded with symmetrical SRRs. (**b**) Lumped element circuit model. (**c**) Photograph of the embroidered design. (**d**) S-parameter responses of the EM simulation, equivalent circuit model, and measurement of embroidered transmission line loaded with two symmetric SRRs.

A second study case was considered for the implementation of an MTM transmission line with symmetry of SRRs, as shown in Figure 5a, with the aim to enhance the filter performance in some applications. The lumped element equivalent circuit model of the structure considering magnetic coupling between the line and SRRs is depicted in Figure 5b. The extracted parameters are the following: L = 10 nH, C = 5 pF, C_r1_ = 6.3 pF, L_r1_ = 1.65 nH, C_r2_ = 4.03 pF, L_r2_ = 4.3 nH, M = 0.57 nH, M′ = 0.09 nH. 

Figure 5c shows the photograph of the prototype of the fabricated wearable MTM transmission line loaded with two symmetrical SRRs. In this case, an embroidered 40% stitch density satin pattern was used. The considered substrate was felt with thickness h = 1 mm, dielectric constant *ε*_r_ = 1.2, and loss tangent tan *δ* = 0.0013.

As depicted in Figure 5d, the simulated and measured resonance frequencies were in good agreement with the equivalent circuit model at frequencies lower than 2 GHz. The reflection band reaches values lower than S21 < −20 dB with a rejection bandwidth significantly enhanced with regard to the one SRR case, due to the double SRR effect. 

Although the transmission line performance was expected to decrease due to the reduction of the stitch density (in comparison with the previous case), an acceptable attenuation level was obtained in the pass band at UHF frequencies. 

## 5. Effects of Bending

The convenience, robustness, flexibility, and operational reliability of the stitched prototypes for various bending positions are very important, especially when the prototypes are made of textile materials and they are frequently bent due to human body morphology and movements. Therefore, it is necessary to investigate the prototype’s performance characteristics under bending conditions.

**Figure 6:**
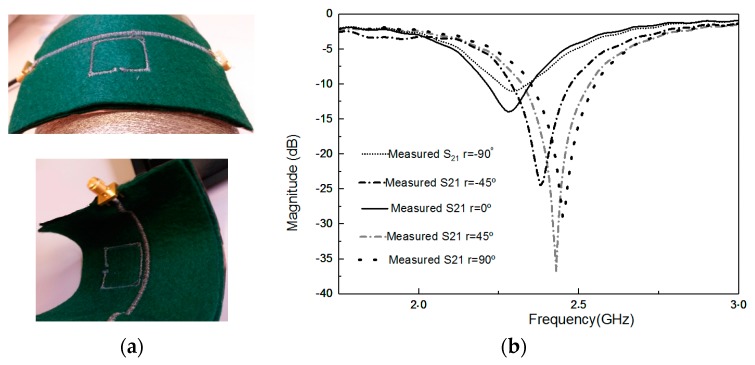
(**a**) Effect of bending with different curved angles. (**b**) Resonant frequency of first prototype on the felt substrate.

S_21_ parameters of an e-textile MTM-SRR under different bending radii were measured. It was observed that due to bending, the equivalent length of the proposed design changed and, hence, there were shifts in the resonant frequency. The more the prototype was bent, the more the resonant length was reduced, and so the resonant frequency got shifted up. This fact was evident from the experimental observations, as shown in Figure 6b. By changing the radius of bending from −90° to 90° (typical human arm radii range), the resonant frequency was shifted up 144 MHz for the felt substrate case.

## 6. Conclusions

In this paper, the utilization of embroidered MTM e-textiles for implementing a controllable transmission line was presented for wearable applications. The proposed design was a fully-embroidered conductive thread transmission line loaded with a conductive yarn SRR on a felt fabric substrate. The comparisons of full 3D electromagnetic simulations and measurements, as well as the analysis of equivalent lumped and distributed circuit models, were studied, achieving a significant degree of agreement. Experimental rejection level values higher than 30 dB were achieved in compact coupled structures and a significant degree of design control in terms of bandwidth was achieved for moderate stitch density embroidery patterns, therefore minimizing the metallic thread cost. Additionally, the effect of bending the manufactured e-textiles MTM transmission line had been tested and a maximum 144 MHz resonance frequency shift in terms of typical bending parameters due to conformal values relative to the human body shape had been obtained. The validated results confirm embroidered MTM e-textiles as a useful technique to control and filter the propagation of UHF signals on wearable applications. 

## References

[1] Islam M.M., Islam M.T., Samsuzzaman M., Faruque M.R.I., Misran N., Mansor M.F. (2015). A Miniaturized Antenna with Negative Index Metamaterial Based on Modified SRR and CLS Unit Cell for UWB Microwave Imaging Applications. Materials.

[2] Hinojosa J., Saura-Ródenas A., Alvarez-Melcon A., Martínez-Viviente F.L. (2018). Reconfigurable Coplanar Waveguide (CPW) and Half-Mode Substrate Integrated Waveguide (HMSIW) Band-Stop Filters Using a Varactor-Loaded Metamaterial-Inspired Open Resonator. Materials.

[3] Islam M.M., Islam M.T., Faruque M.R.I., Samsuzzaman M., Misran N., Arshad H. (2015). Microwave Imaging Sensor Using Compact Metamaterial UWB Antenna with a High Correlation Factor. Materials.

[4] Pendry J.B., Holden A.J., Robbins D., Stewart W. (1999). Magnetism from conductors and enhanced nonlinear phenomena. IEEE Trans. Microw. Theory Tech..

[5] Stancil D.D. (1993). Theory of Magnetostatic Waves.

[6] Tay Z.J., Soh W.T., Ong C.K. (2018). Observation of electromagnetically induced transparency and absorption in Yttrium Iron Garnet loaded split ring resonator. J. Magn. Magn. Mater..

[7] Tsolis A., Whittow W.G., Alexandridis A.A. (2014). Embroidery and Related Manufacturing Techniques for Wearable Antennas: Challenges and Opportunities. Electronics.

[8] Seager R., Chauraya A., Vardaxoglou J. (2008). Fabric antennas integrated with metamaterials. Metamaterials.

[9] Mirotznik M.S., Yarlagadda S., McCauley R., Pa P. (2012). Broadband electromagnetic modeling of woven fabric composites. IEEE Trans. Microw. Theory Tech..

[10] Michalak M., Kazakevičius V., Dudzińska S., Krucińska I., Brazis R. (2009). Textiles Embroidered with Split-Rings as Barriers Against Microwave Radiation. Fibres Text. East. Eur..

[11] Hong J.-S.G., Lancaster M.J. (2004). Microstrip Filters for RF/Microwave Applications.

[12] Ikalainen P.K., Matthaei G.L. (1987). Wide-Band, Forward-Coupling Microstrip Hybrids with High Directivity. IEEE Trans. Microw. Theory Tech..

